# 3D multiple immunoimaging using whole male organs in rice

**DOI:** 10.1038/s41598-022-19373-4

**Published:** 2022-09-14

**Authors:** Saori Araki, Hinako Tamotsu, Reina Komiya

**Affiliations:** 1 Science and Technology Group, Okinawa Institute of Science and Technology Graduate University (OIST), 1919-1 Tancha, Onna-Son, Okinawa, 904-0495 Japan; 2 PRESTO, Japan Science and Technology Agency (JST), 4-1-8 Honcho, Kawaguchi, Saitama 332-0012 Japan

**Keywords:** Cell biology, Molecular biology, Plant sciences

## Abstract

Spatiotemporal regulation of proteins and RNAs is essential for the precise development of reproductive tissues in many organisms. The anther, a prominent part of the male reproductive organ in plants, contains several somatic cell layers named the anther wall and, within it, the germ cells. Here, we successfully developed a simple 3D organ-immunoimaging technique for rice anthers, which distinguishes each individual cell from the four somatic cell layers and germ cells without the need for transformation, embedding, sectioning, or clearing. The 3D immunostaining method is also applicable to the intracellular localization of meiosis-specific proteins in meiocytes, as exemplified by MEL1, a germ cell-specific ARGONAUTE in the cytoplasm, and ZEP1, a pachytene marker on meiotic chromosomes. Our 3D multiple immunostaining method with single-cell and intracellular resolution will contribute to a comprehensive organ-level elucidation of molecular mechanisms and cellular connectivity.

## Introduction

The development of three-dimensional (3D) organ imaging at subcellular and single-cell resolution is essential for a comprehensive understanding of molecular mechanisms because the developmental mechanisms in many organisms involve precise spatiotemporal regulation^1^. Reproductive organs consist of somatic cells and germ cells in multicellular eukaryotes. Site-specific regulation of three ARGONAUTE (AGO) proteins, which function as the core silencing machinery, between soma and germ is essential for development of the *Drosophila* ovary^2–4^. In addition to such spatial control, silencing or transcription factors also mediate cell connectivity from soma to germ in the plant reproductive organs, as previously reported^5–9^. Hence, the development of whole-organ immunohistochemical techniques can also help to elucidate the developmental molecular mechanisms and cellular connectivity between soma and germ, distinguishable at the single-cell level, in animals and plants.

**Figure 1:**
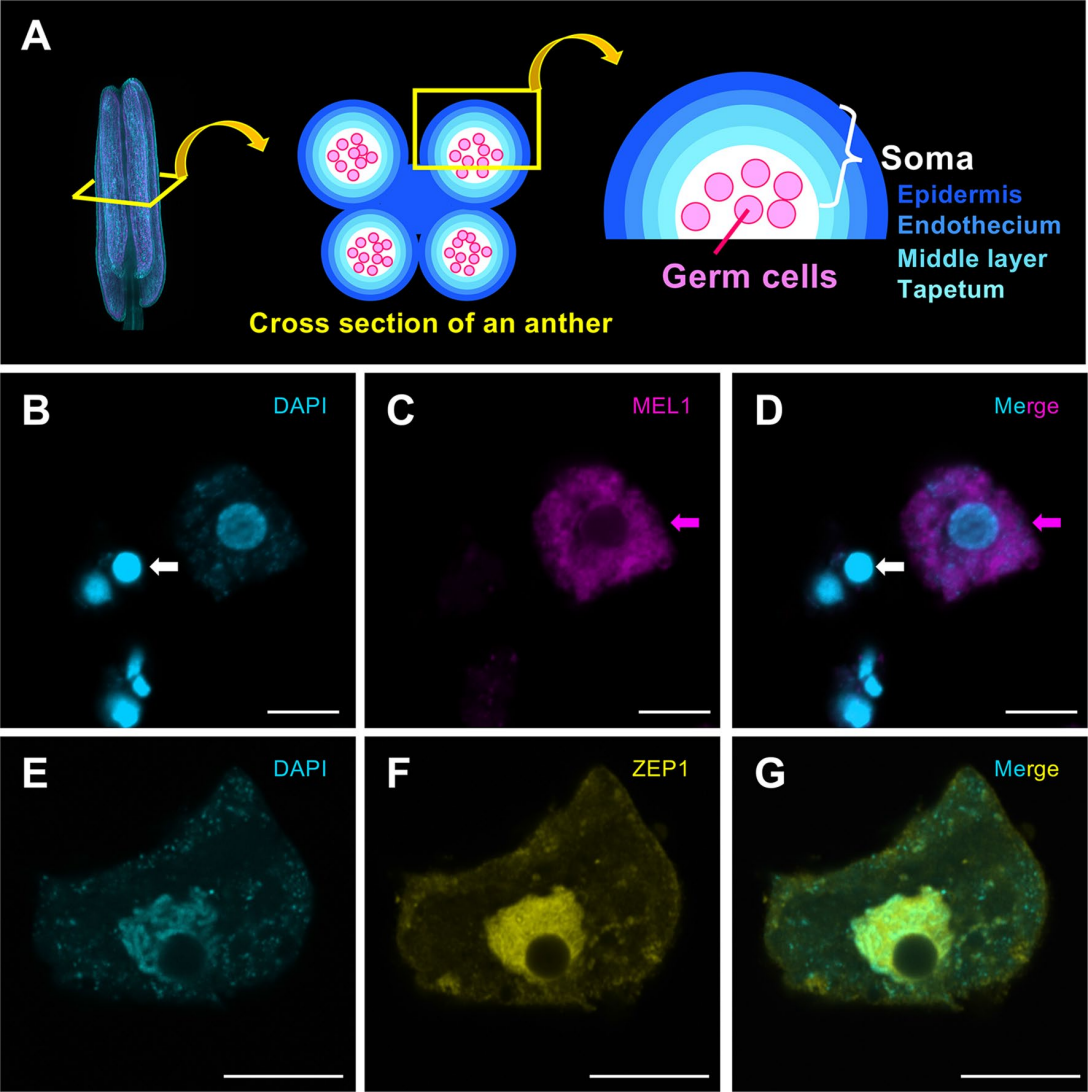
Immunostaining using pollen mother cells for anti-MEL1 and meiocytes for anti-ZEP1. (**A**) Schematic diagram of an anther. The image on the left shows an anther stained with SCRI Renaissance 2200. The anther consists of four locules (middle), and a cross-section of a single locule demonstrates the four somatic anther wall layers (right), including the epidermis (Ep), endothecium (En), middle layer (Ml), and tapetum (Ta), which enclose the germ cells. (**B**–**D**) MEL1 localization is restricted to the cytoplasm (magenta). White arrows indicate a single somatic cell from the anther wall, although its origin from a specific somatic cell type (Ep, En, Ml, or Ta) remains unknown. Magenta arrows indicate a pollen mother germ cell at the premeiotic stage. (**E**–**G**) Indirect fluorescence signals of ZEP1 are enriched on the meiotic chromosomes (yellow). Cyan signals indicate DAPI staining (**B** and **E**). Merged images of (**B**) and (**C**), and (**F**) and (**G**), respectively (**D** and **G**). Scale bars, 10 μm.

The anther is a major part of the male reproductive organ in plants, and consists of germ cells, also known as the pollen, and the anther wall, which comprises three to four outer somatic layers surrounding the germ cells (Fig. 1A). To investigate the structure of tissues and to distinguish each single cell, morphological imaging is beneficial in tandem with histochemical staining. However, morphological analysis of structures such as the anther requires many steps, including fixation of tissue, embedding in resin, sectioning, and staining^10,11^. Our recent histochemical imaging study enabled visualization of the 3D structure of entire rice anthers using light sheet microscopy^12^. This method can also be used to reveal the internal structure of the anthers, indicating that morphological analysis is suitable for detecting developmental defects in mutants.

To investigate the localization of proteins and to elucidate the molecular mechanisms involved, imaging using fluorescent proteins is better established than immunostaining in plants^13,14^. Notably, Schnittger and colleagues have successfully developed live-cell imaging to observe meiotic progression using the buds of reporter lines in *A. thaliana*^15,16^. However, these methods require an additional transformation step to generate plants with the target protein fused to a fluorescent protein. In contrast, immunohistochemical methods using meiocytes (germ cells undergoing meiosis) are established^17–19^, although they include a cellulase treatment step that disrupts the anther wall, thus precluding the identification of which somatic layer an individual cell is derived from^20^. Meanwhile, current immunoimaging techniques, which can detect epigenetic marker proteins in leaves, involve clearing steps^21^ and are thus not suitable for reproductive tissues in plants. Accordingly, 3D histological immunoimaging using whole anthers is potentially of great benefit for determining spatiotemporal regulation during plant reproduction.

Here, we successfully developed a 3D anther-immunostaining method to visualize intracellular protein localization, as illustrated by the cytoplasmic MEIOSIS ARRESTED AT LEPTOTENE (MEL1) and the filamentous ZEP1, and to distinguish each individual cell in the four somatic cell layers in rice. The advantages of our simple method are that it can reveal the subcellular localization of multiple proteins simultaneously in the four somatic cell layers and germ cells, and that it eliminates the need for transformation, embedding, cross-sectioning, and clearing.

## Results

### Production and evaluation of antibodies

Anther development is approximately classified into four stages: premeiosis, early meiosis, meiotic division, and microspore (Supplementary Table S1)^22^. Meiosis is an essential phenomenon in which genetic information is inherited by the subsequent generation^23^. ZEP1 is a component of transverse filaments of the rice synaptonemal complex^24^. The mutant of MEL1, a reproductive AGO, exhibits a defect of ZEP1 elongation on meiotic chromosomes, resulting in the arrest of homologous chromosome pairing^20,25^. Thus, MEL1 is required for meiotic progression during early meiosis in rice via ZEP1 elongation on meiotic chromosomes. First, we generated antibodies against MEL1 and ZEP1 as a germ cell marker and a pachytene marker, respectively, to develop the 3D immunoimaging method using whole rice anthers (see “Methods”). Automated western analysis using Wes, with MEL1 and ZEP1 antibodies, revealed that MEL1 and ZEP1, with the predicted molecular masses, were expressed at the early meiotic stage, where the anthers are 0.6–0.7 mm long (Supplementary Fig. S1). Moreover, we performed immunostaining using a previously reported method^20^ and detected these proteins using pollen mother cells for MEL1 and meiocytes for ZEP1. MEL1 was enriched in the cytoplasm in pollen mother cells (Fig. 1B–D), while ZEP1 was restricted to the meiotic chromosomes at the progression of homologous chromosome synapsis during early meiosis, as previously reported (Fig. 1E–G)^20,24^. Therefore, these MEL1/ZEP1 antibodies are suitable meiocyte/meiotic landmarks in 3D multiple immunostaining.

### Immunostaining using whole anthers

**Figure 2:**
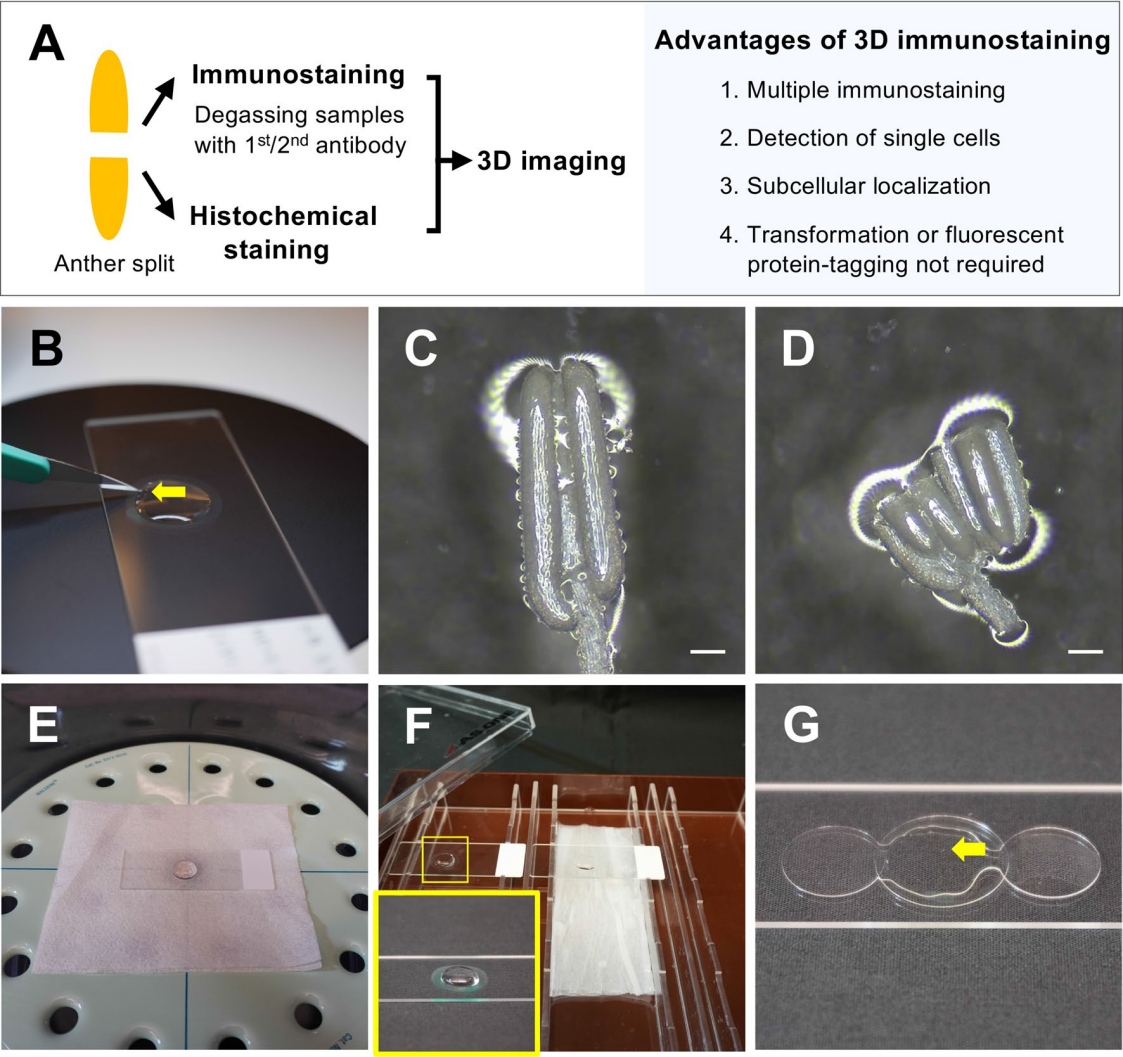
Equipment used for 3D multiple immunostaining and splitting anthers. (**A**) Flow chart and advantages of the 3D multiple immunostaining method. (**B**) Anthers being split using a scalpel (arrow) on a MAS-coated slide under a stereo microscope. (**C**) A 4% PFA-fixed anther in distilled water before the split. (**D**) The two parts of an anther after the split. Scale bars, 100 μm. (**E**) Samples with primary/secondary antibody solution in a vacuum desiccator. (**F**) Samples without coverslips placed in a humidified box for primary/secondary antibody incubations. Yellow-lined square shows an enlarged image of the antibody treatment on the slide. (**G**) Mounting of samples. Two additional coverslips were placed on either side of the sample to maintain the structure of anthers without crushing them. Yellow arrow indicates the split anther.

Splitting anthers and degassing prior to immunostaining with primary and secondary antibodies are indispensable steps in 3D multiple immunoimaging (Fig. 2A). Anther lengths correlate with the anther development stages, including somatic anther wall and germ cell development (Table S1). We isolated 0.6–0.7-mm anthers from 4% paraformaldehyde (PFA)-fixed inflorescences during early meiosis, when homologous chromosome synapsis occurs. The four somatic layers of the anther wall (epidermis, endothecium, middle layer, and tapetum) are formed, surrounding the meiocytes, at the early meiosis stage (Fig. 1A). For the antibodies to penetrate the whole anthers, the PFA-fixed anthers were transferred to distilled water on MAS-coated microscope slides and split into two using a scalpel under a stereomicroscope, taking care to not collapse their structure (Fig. 2B–D). This process of anther splitting contributes to the success of 3D multiple immunoimaging.

After the splitting and blocking steps, the anthers were immersed in primary MEL1 and ZEP1 antibody solutions, 1/500 with 3% bovine serum albumin (BSA) in PME buffer (see “Methods” for details). Next, degassing in a vacuum desiccator (Fig. 2E) for the primary and secondary immunostaining steps is required for antibody penetration into the whole anther. The uncovered slides were transferred to a humidified box and incubated overnight at 4 °C (Fig. 2F). After washing three times, the slides were immersed in secondary antibody solution, and degassed. The slides were incubated in a dark humid chamber for 2 h at 25 °C and then incubated overnight at 4 °C. After washing, the samples were stained with 4′,6-diamidino-2-phenylindole (DAPI) and mounted in ProLong Gold; two coverslips were placed on either side of the samples to avoid crushing the anther locules (Fig. 2G).

### Visualization of the 3D anther immunostaining

**Figure 3:**
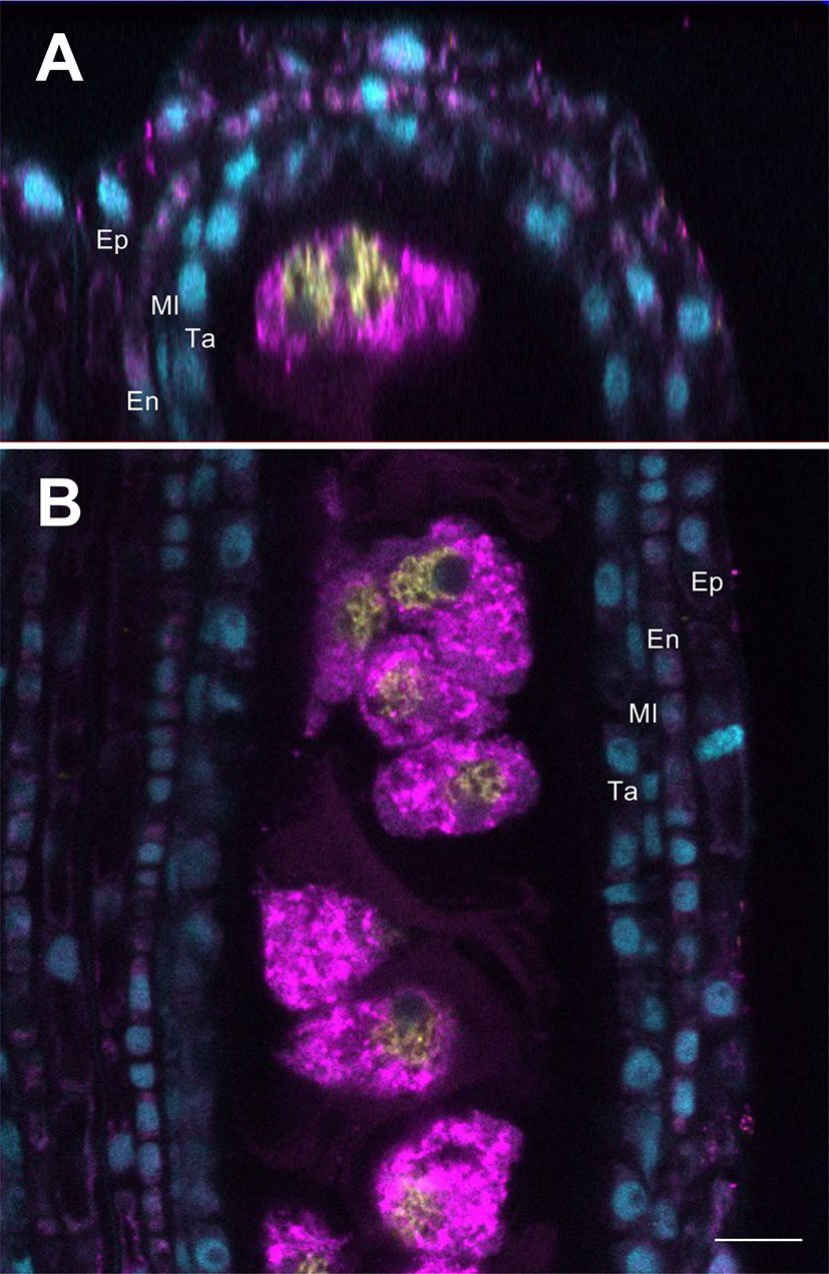
Cross and longitudinal sections for 3D anther immunoimaging using a confocal microscope. (**A**) A cross-section (X section) of the 3D anther immunoimaging, in which 0.65-mm anthers from the early meiosis stage were used. (**B**) A longitudinal section (Z section) of the 3D anther immunoimaging. The four somatic layers, epidermis (Ep), endothecium (En), middle layer (Ml), and tapetum (Ta), were observed. The cytoplasmic localization of MEL1 (magenta) and nuclear localization of ZEP1 (yellow) fluorescence signals were observed in meiocytes surrounded by the somatic anther wall. Cyan signals indicate DAPI staining. Scale bar, 10 μm.

We captured continuous image data of the anther, consisting of 101 slices with 0.5-μm intervals from the outer epidermis to the inner meiocytes in longitudinal Z sections, using an LSM 880 microscope (Carl Zeiss), and created a movie by stacking these images using Imaris 9 (Movie 1). We thus successfully identified the positions of all cells, and each of the five cell types including anther wall and germ cells, of the anther. It is also possible to distinguish the four somatic cell layers in X sections, which are cross-sections of anthers (Fig. 3A), as well as Z sections (Fig. 3B). DAPI signals were also detected in all somatic anther wall layers, namely the epidermis, endothecium, middle layer, and tapetum. Moreover, the localization of MEL1 and ZEP1 was restricted to the meiocytes. In particular, MEL1 was detected in the cytoplasm, while ZEP1 was identified on the meiotic chromosomes, indicating that 3D immunostaining is a multiplex imaging method that allows for differentiating between the four somatic cell layers and the germ cells, and for determining the subcellular localization of multiple proteins.

We also performed 3D immunoimaging using 0.5-mm anthers at premeiotic stages to detect heterochromatin formation. Dot signals of dimethyl histone H3 (Lys9) were observed in the nucleus of pollen mother cells (Supplementary Fig. 2A–C), indicating that this 3D immunostaining method can be used for other anther developmental stages and against general proteins, not only meiosis-specific factors. Furthermore, these signals can also be detected in anthers of the same stage that were fixed 1 year previously (Supplementary Fig. 2D–F).

Next, we investigated 3D histochemical staining using whole anthers. We also split the PFA-fixed anthers, which are 0.5 mm long in the early meiosis stage, into two parts, and treated them with propidium iodide (PI) to stain DNA/RNA. We detected fluorescence signals in the four anther wall layers and in pollen mother cells (Supplementary Fig. S3). Consequently, these methods using the split anthers are effective for 3D immunostaining and 3D histochemical staining (Fig. 2A).

### Single-cell morphology and MEL1/ZEP1 subcellular localization using the whole-anther immunostaining method

**Figure 4:**
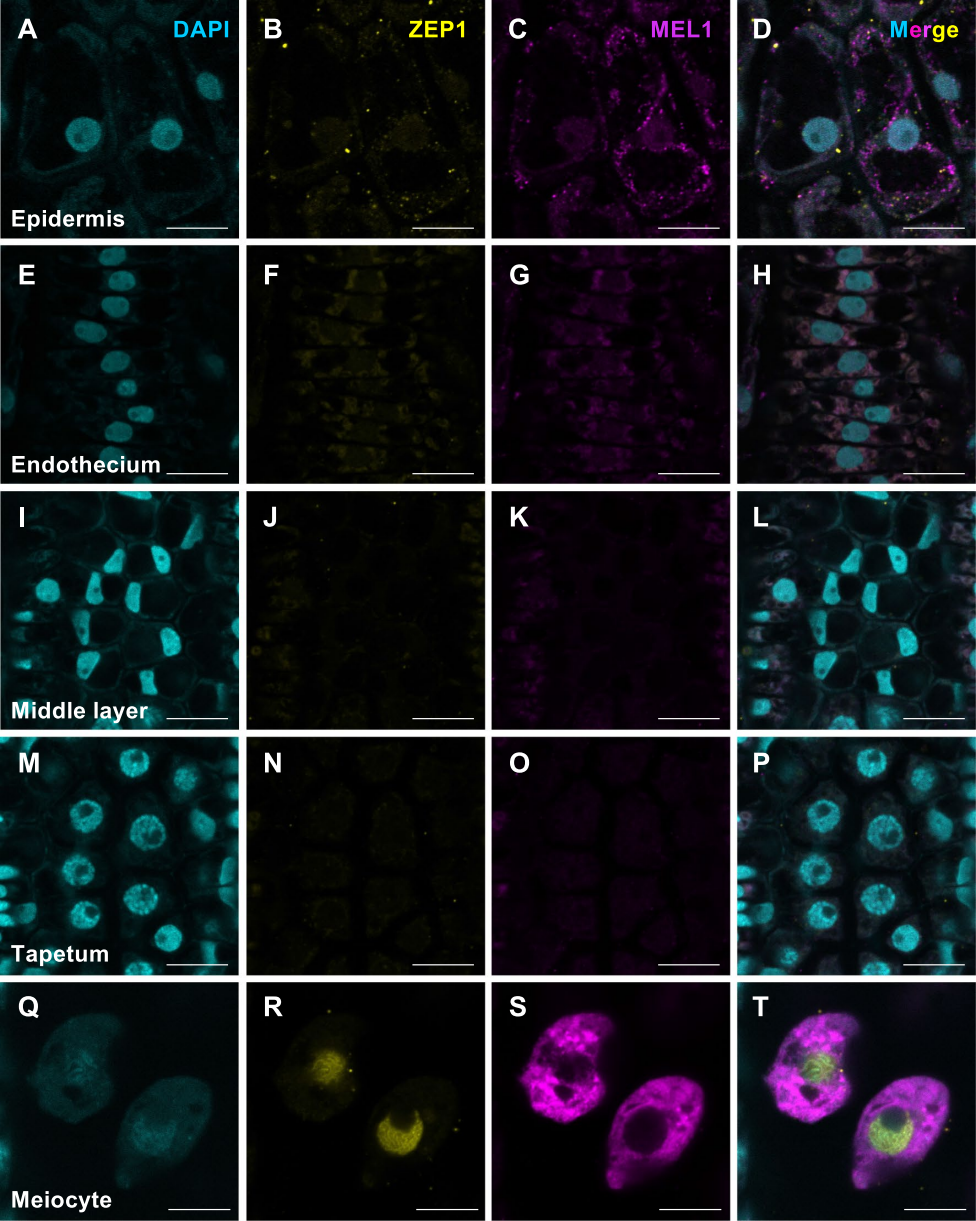
Single-cell morphology and MEL1/ZEP1 subcellular localization using the whole-anther immunostaining method. (**A**,**E**,**I**,**M**,**Q**) DAPI signals (cyan) in 3D multiple immunoimaging using 0.65-mm-long whole anthers at early meiosis. (**B**,**F**,**J**,**N**,**R**) 3D multiple immunoimaging to detect ZEP1 (yellow). (**C**,**G**,**K**,**O**,**S)** 3D multiple immunoimaging to detect MEL1 (magenta). (**D**,**H**,**L**,**P**,**T**) Merged images of (**A**–**C**,**E**–**G**,**I**–**K**,**M**–**O**,**Q**–**S**), respectively. This 3D immunostaining method enables us to distinguish cell types comprising the epidermis (**A**–**D**), endothecium (**E**–**H**), middle layer (**I**–**L**), tapetum layer (**M**–**P**), and meiocytes (**Q**–**T**). Scale bars, 10 μm.

Finally, we explored the structure of single cells in each cell layer and in meiocytes of rice anthers during early meiosis using the 3D multiple immunoimaging method. The epidermis cells were the largest in the anther wall layers (Fig. 4A–D), and the endothecium cells exhibited transversally elongated cell shapes (Fig. 4E–H). The nuclei of middle layer cells showed a characteristic flattened structure, whereas those in the tapetum layer were round (Fig. 4I–P). MEL1 was primarily present in the cytoplasm in meiocytes (Fig. 4S,T), whereas ZEP1 was detected in the nuclear region (Fig. 4R,T). Notably, the filamentous signals of ZEP1 were detected on the meiotic chromosomes, which coincide with the DAPI-stained meiotic chromosomes (Fig. 4Q,R,T). The contrasting intracellular localization of cytoplasmic MEL1 and nuclear filamentous ZEP1 demonstrates that the simple 3D multiple immunoimaging distinguishes the subcellular localization in each individual cell of the anther.

We successfully identified the positions of all cells, and all cell types, of the anther (Movie 1 and Fig. 4). Accordingly, it would be possible to compare the intensity of each fluorescence signal quantitatively in subcellular localization and in cell-type localization, including nucleus/cytoplasm and germ/soma.

## Discussion

In this study, we developed a 3D imaging method that included histochemical staining and multiple immunostaining, for the study of whole anthers, which enabled us to distinguish between single cells among the four somatic cell types in the anther without sectioning the anthers. Furthermore, it was possible to clearly detect filamentous signals of ZEP1 on the meiotic chromosomes in addition to the specific subcellular localization of MEL1 and ZEP1, indicating that 3D immunoimaging methods can clarify the organellar localization of proteins in each individual cell.

iTOMEI clearing is a powerful method to reduce the autofluorescence of plant organs^14,26^. It was also applicable for the 3D histochemical staining with PI in this study (Supplementary Fig. S3). However, when we performed the iTOMEI clearing after 3D immunoimaging of anthers, it was difficult to detect the fluorescence signals, suggesting that the clearing method is not compatible with immunostaining using Alexa Fluor 567 and Alexa Fluor 678. Because the autofluorescence of anthers is considerably weaker than that of leaves, the clearing step is not required for 3D anther immunoimaging. Using this simple immunostaining method, it was possible to observe fluorescence signals in the middle of an anther locule, around 50 μm from the anther surface, allowing us to detect all four types of somatic anther wall cells as well as germ cells in anthers without clearing.

One of the advantages of this immunoimaging method is that it does not require the transformation step to generate plants with fluorescent proteins. There is also no need for resin embedding, sectioning of samples, or clearing during 3D multiple immunoimaging (Fig. 2A). The step of anther splitting is important for successful 3D multiple immunostaining of anthers. All procedures are performed directly on the same slide, with no need for a slide rack or a staining jar for treatment and washing. Moreover, multiple immunoimaging could be performed using several antibodies to elucidate the localization of more proteins simultaneously and can achieve high resolutions to investigate subcellular localization in single cells. The remarkable feature of a live-cell imaging procedure using buds in *A. thaliana* is that it enables sequential observation over the timescale of meiosis^16^. Addressing the temporal element of spatiotemporal regulation lays the groundwork for the next phase in 3D organ immunoimaging. In contrast, 3D organ immunostaining has the advantage that samples can be stored for at least 1 year, allowing observation at any time (Supplementary Fig. S2), while the observation timing of 4D imaging is sample-dependent because it uses non-fixed samples^27^.

Improving this 3D immunoimaging system may facilitate the development of 3D fluorescence in situ hybridization using the whole anther to detect the subcellular localization of mRNAs, long non-coding RNAs, and small RNAs in the near future. A large number of 21- and 24-nucleotide small RNAs are produced specifically in the anther and are loaded onto reproductive AGOs^20,28,29^. These anther small RNAs are spatiotemporally classified into two groups, premeiotic small RNAs in the somatic outer layers and meiotic small RNAs in the inner layers/meiocytes, during anther development^22,30^. Furthermore, 24-nucleotide small RNAs of the anther reportedly move from the tapetum to the meiocytes^21,31^, indicating the possibility of cell-to-cell interaction via small RNAs as mobile signals in anthers. Hence, the method has potential applicability in simultaneously monitoring the localization of multiple proteins and RNAs. The 3D organ immunoimaging developed here should enable comprehensive organ-level elucidation of molecular mechanisms, dynamics, and cellular communications, and will also be applicable to other living systems.

## Methods

### Plant materials and growth conditions

Rice (*Oryza sativa* L., subspecies Japonica, cultivar Nipponbare) was used in this study. We obtained seeds of Nipponbare from the National Institute of Genetics, Japan. The Nipponbare genome has been sequenced by the International Rice Genome Sequencing Project and is available in the Rice Annotation Project Database (RAP-DB; https://rapdb.dna.affrc.go.jp/).

Plants were grown in growth chambers at 70% humidity with a daily cycle of 14 h of light at 29.5–30 °C and 10 h of darkness at 25 °C for 40 days and then transferred to short-day conditions, with 10 h of light and 14 h of darkness, to promote the induction of the reproductive stage and to adjust the sampling stages.

### MEL1 and ZEP1 antibody generation and H3K9dM antibody

Two oligopeptides, MEL1-1 (CVYGAPMPAAHHQGAYQ) and MEL1-2 (GQAVAREGPVEVRQLPKC), were used to raise antibodies in rabbits (Cosmo Bio). The rabbit antisera were purified using affinity chromatography (Cosmo Bio).

An N-terminal region of ZEP1 cDNA was amplified by PCR using the primer pair 5′-gacaagcttgcggccATGCAGAAGCTGGGTTTATC-3′ and 5′- tgctcgagtgcggcctgTTCAGCAGATCTAGAATCCTCC-3′, where lower-case letters indicate vector sequences for cloning the ZEP1 amplicon into pET24(+) (Novagen, 69772) using an infusion system. The transformation of BL21 (DE3) pLys, protein purification, and immunization of rats were performed commercially (Scrum). To detect the localization of histone 3 K9 dimethylation, an anti-dimethyl histone H3 (Lys9), mouse monoclonal antibody (FUJIFILM, 308-32361) was used for the 3D immunoimaging, Supplementary Fig. 2.

### Wes

Wes is automated western analysis using capillary rather than gel blotting. Results are shown in a digital image similar to a western blot and in graphs using raw data. Proteins were extracted from 0.6 to 0.7-mm long anthers. The anthers were ground and mixed with an extraction buffer (150 mM NaCl, 50 mM Tris–HCl (pH 7.5), 0.1% Tween 20, 10% glycerol, 5 mM DTT, 1 mM Pefabloc SC (Roche), 1× cOmplete Protease Inhibitor Cocktail). After two rounds of centrifugation (5800*g* for 10 min at 4 °C and 20,400*g* for 10 min at 4 °C) and removal of the debris, total proteins were extracted. MEL1 and ZEP1 antibodies (1/20 dilution) were used for the initial immune reactions.

### Paraformaldehyde fixation

Anthers were fixed as previously described^12^. PFA fixative was prepared fresh immediately before use, and 4% PFA (Alfa Aesar) was added in 1× PMEG buffer (50 mM PIPES (Dojindo Molecular Technologies), 10 mM EGTA, 5 mM MgSO_4_·7H_2_O, 4% glycerol, 0.2% DMSO; pH 6.8). The inflorescences in the PFA fixative were degassed at 0.09–0.1 MPa for 20 min on ice (Supplementary Fig. S4), and this step was repeated three more times. The samples were incubated for 100 min at 25 °C and washed in 1× PMEG buffer for 20 min at 25 °C; this wash step was repeated five more times. The fixed inflorescences can be stored at 4 °C for 1 year.

### Immunostaining using pollen mother cells for antibody estimation

To release the meiocytes, anthers from fixed inflorescences were incubated in an enzyme cocktail containing 2% cellulase Onozuka-RS (Yakult Honsha), 0.3% pectolyase Y-23 (Kikkoman), and 0.5% Macerozyme-R10 (Yakult Honsha) in PME buffer (50 mM PIPES, 5 mM EGTA, and 5 mM MgSO_4_; pH 6.9)^17^ on a MAS-coated microscope slide, for 1 min at 25 °C. The anthers were washed with PME, squashed in distilled water using a needle to release the meiocytes, and incubated at 25 °C for 30 min. The meiocytes were then blocked with 3% BSA in PME for 60 min and incubated at 4 °C overnight with rabbit anti-MEL1 or rat anti-ZEP1 antibody, diluted 1/500 with 3% BSA in PME. After washing three times with PME for 5 min, the slide was incubated in a dark chamber for 3 h at 25 °C with Goat anti-Rabbit IgG (H + L) Highly Cross-Adsorbed Secondary Antibody, Alexa Fluor 488 (Invitrogen, A11034) and Goat anti-Rat IgG (H + L) Cross-Adsorbed Secondary Antibody, Alexa Fluor 647 (Invitrogen, A21247), diluted 1/200 with 3% BSA/PME, followed by three washes with PME for 5 min each. The MEL1 sample was then mounted in Vectashield mounting medium with DAPI (Vector Laboratories, H-1200), and images were captured using an LSM 780 microscope (Carl Zeiss). ZEP1 samples were incubated for 15 min at 25 °C in DAPI (Sigma, MBD0015), followed by washing three times with PME buffer. Samples were mounted in ProLong Gold antifade reagent (Invitrogen, P10144) and images were captured using an LSM 880 microscope (Carl Zeiss).

### Immunostaining using whole mounts of anthers

Anthers were retrieved from 4% PFA-fixed inflorescences in PME buffer under a stereomicroscope. Next, the anthers were transferred to distilled water in a circle drawn with a PAP pen (Daido Sangyo) on a MAS-coated microscope slide (Matsunami Glass) and split into two parts using a scalpel (see Fig. 2B–D); the samples were incubated for 30 min at 25 °C. After blocking with 3% BSA in PME for 60 min at 25 °C, the cut anther samples were immersed in primary antibody solution (rabbit anti-MEL1, rat anti-ZEP1 or mouse anti-dimethyl H3 diluted 1/500 with 3% BSA in PME), placed in a vacuum desiccator (Nalgene) and degassed at 0.05 MPa for 2 min at 25 °C, which was repeated four more times, and incubated overnight at 4 °C. After washing three times with PME for 5 min, the slide was placed in secondary antibody solution, anti-Rabbit IgG, Alexa Fluor 568 (Invitrogen, A11036), anti-Rat IgG, Alexa Fluor 647 (Invitrogen, A21247) or anti-Mouse IgG, Alexa Fluor 488 (Invitrogen, A11001) diluted 1/200 with 3% BSA in PME, and degassed at 0.05 MPa for 2 min at 25 °C, which was repeated four more times. The slide was incubated in a dark humid chamber for 2 h at 25 °C and then incubated overnight at 4 °C. After washing three times with PME buffer for 5 min, the samples were incubated for 15 min at 25 °C in DAPI (Sigma, MBD0015), followed by three washes with PME buffer. Samples were mounted in ProLong Gold antifade reagent (Invitrogen, P10144) with coverslips, which are 0.13–0.17 mm in thickness (MATSUNAMI Micro Cover Glass 13 mm No. 1).

### Visualization of the 3D immunostaining of entire anthers

Images were captured using an LSM 880 microscope (Carl Zeiss) under the following conditions: 40× (1.3 oil) Plan Apochromat lens (Fig. 3 and Movie 1) and 63× (1.4 oil) Plan Apochromat lens (Fig. 4) for detection; 405, 561, and 633 nm laser lines for DAPI, Alexa Fluor 568, and Alexa Fluor 647 excitation; and 410–483 nm (DAPI), 571–633 nm (Alexa Fluor 568) and 638–755 nm (Alexa Fluor 647) filter emission. The images and animation were created using ZEN (Carl Zeiss) or Imaris 9 (Bitplane AG) software (Figs. 3, 4; Movie 1).

### 3D histochemical staining of whole anthers and imaging

Anthers fixed in 4% PFA were split into two parts as described above. The anthers were stained with PI for 15 min at 25 °C, and then transferred to 20% iTOMEI solution (20% caprylyl sulfobetaine in 100 mM sodium phosphate buffer) and incubated for 10 min at 25 °C. The samples were next transferred to 50% iTOMEI solution (50% caprylyl sulfobetaine in 100 mM sodium phosphate buffer) and incubated for 10 min at 25 °C. Finally, the samples were transferred and incubated in 70.4% iTOMEI (70.4% iohexol in PBS) for 1 h at 25 °C^14^, and mounted with 70.4% iTOMEI. Images were captured with a confocal microscope (LSM 880; Carl Zeiss) under the following conditions: 40× (1.3 oil) Plan Apochromat lens for detection, 561 nm laser lines for PI excitation, and 571–633 nm filter emission. The images were created using ZEN (Carl Zeiss) (Supplementary Fig. S3).

### Ethics approval and consent to participate

The experimental research reported here complies with relevant institutional, national, and international guidelines and legislation.

## Supplementary Information

Supplementary Information 1.

Supplementary Information 2.

Supplementary Movie 1.

Supplementary Figures.

Supplementary Table S1.

Supplementary Movie 1 legend.

## Data Availability

*MEL1*: Os0 3g0800200; *ZEP1*: Os04g0452500. RAP-DB (https://rapdb.dna.affrc.go.jp/).
