# Supplementary material for: 3D multiple immunoimaging using whole male organs in rice
Source: Sci Rep. 2022 Sep 14;12:15426. doi: 10.1038/s41598-022-19373-4 (PMC9475021; doi:10.1038/s41598-022-19373-4)
Supplement: Supplementary file 1 — Supplementary Information 1. [file 41598_2022_19373_MOESM1_ESM.pdf]

Supplementary Information 1

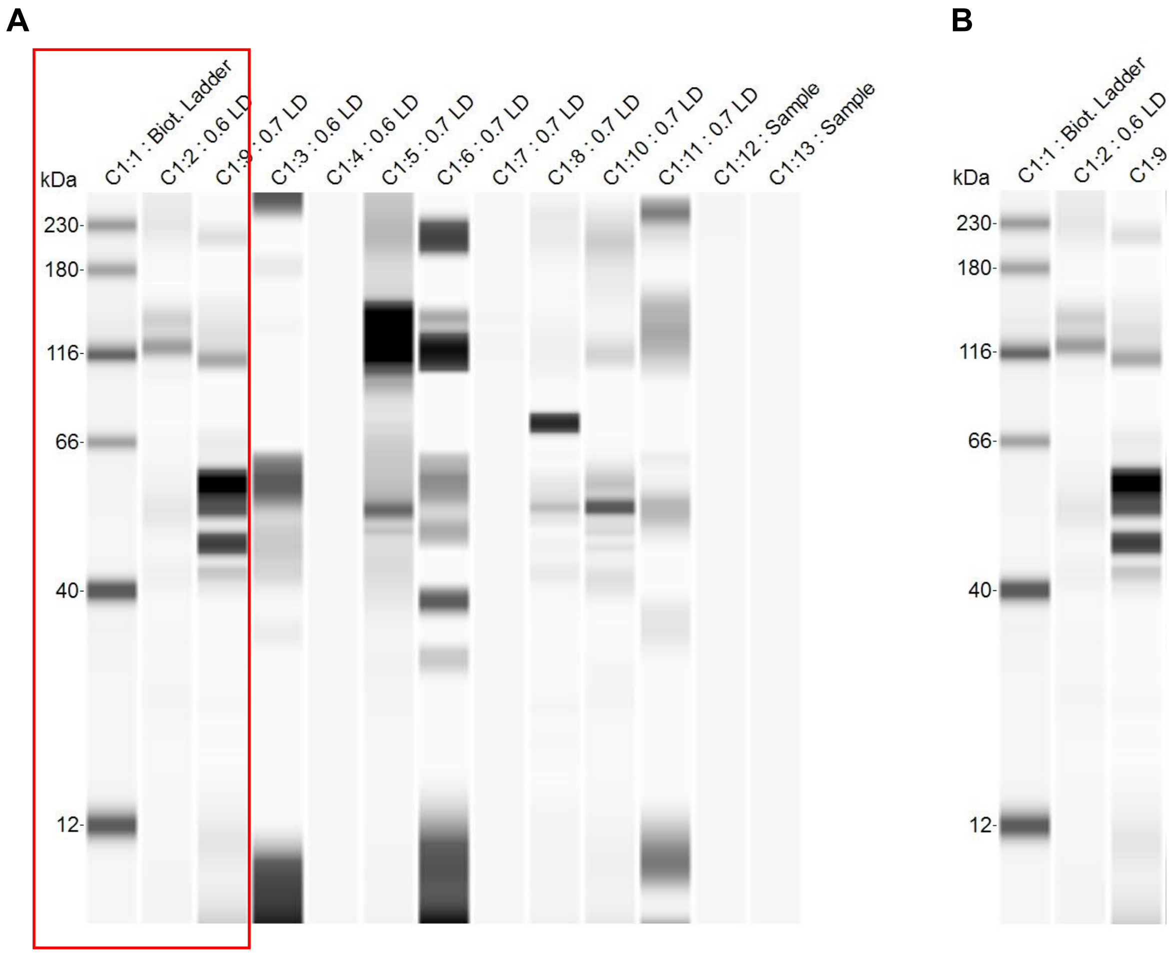

Supplementary Information S1. Raw Wes data for MEL1 and ZEP1 proteins.

(**A**) Full digital image of the Wes data including other samples. The red box highlights the lanes relevant to this study, including those involving MEL1 and ZEP1 antibodies. (**B**) Isolated cropped part of MEL1 and ZEP1 Wes full data in (A), with the full size range included from less than 12 kDa to more than 230 kDa. This image is used as Supplementary Figure 1B.
