# Supplementary figures and images for: 3D multiple immunoimaging using whole male organs in rice
Source: Sci Rep. 2022 Sep 14;12:15426. doi: 10.1038/s41598-022-19373-4 (PMC9475021; doi:10.1038/s41598-022-19373-4)

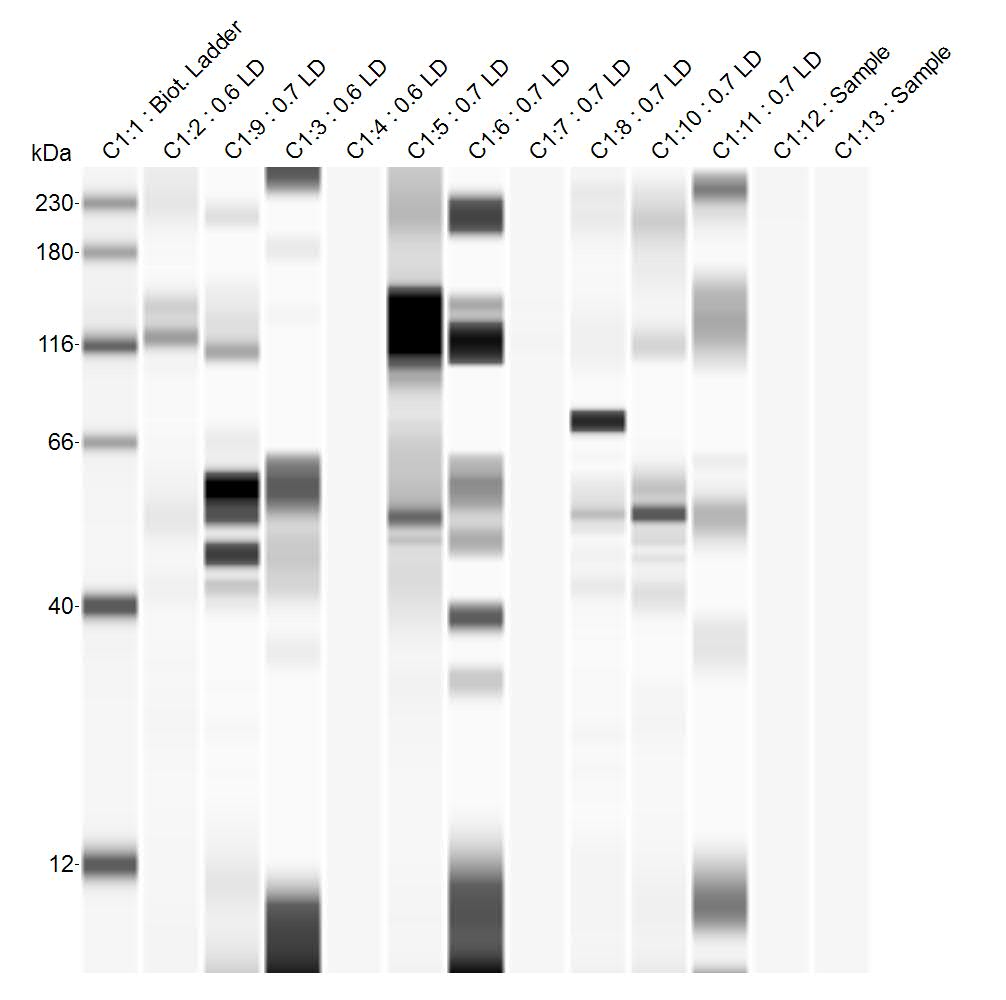

Supplement: Supplementary file 2 — Supplementary Information 2. [file 41598_2022_19373_MOESM2_ESM.jpeg]
