# Supplementary material for: 3D multiple immunoimaging using whole male organs in rice
Source: Sci Rep. 2022 Sep 14;12:15426. doi: 10.1038/s41598-022-19373-4 (PMC9475021; doi:10.1038/s41598-022-19373-4)
Supplement: Supplementary file 4 — Supplementary Figures. [file 41598_2022_19373_MOESM4_ESM.pdf]

Supplementary Figure 1

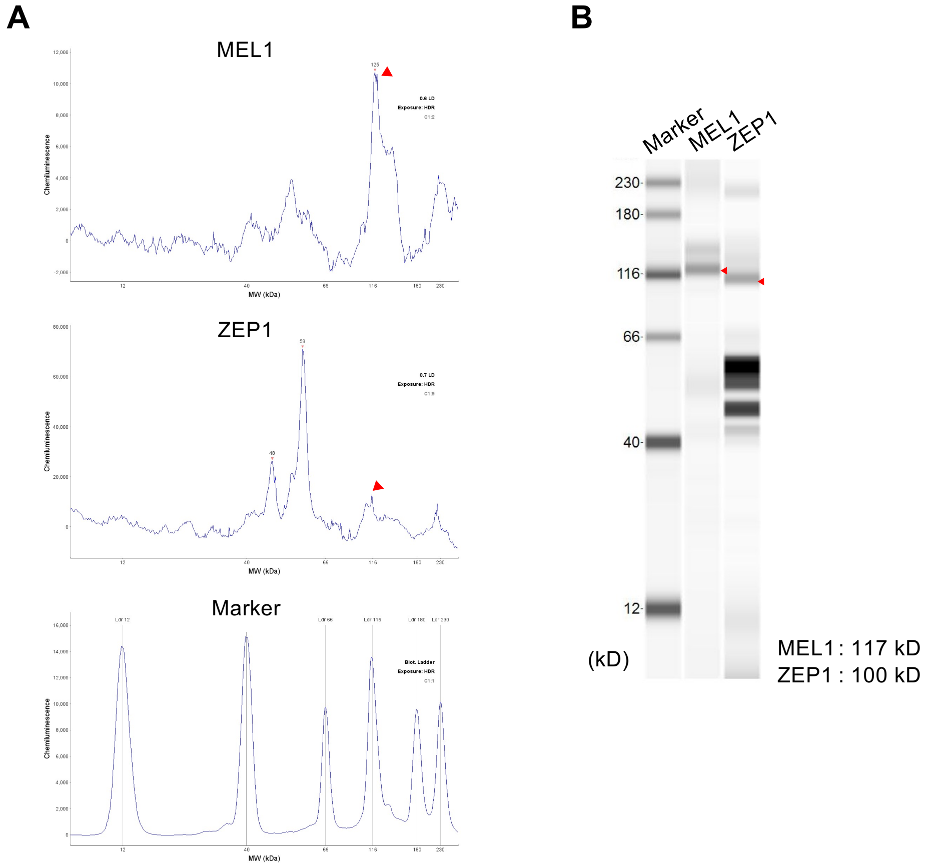

Figure S1. Wes analysis of MEL1 and ZEP1 proteins in anthers during early meiosis.

(**A**) Graphs of raw Wes data. (**B**) Digital images of the Wes data using lanes. Total proteins were extracted from 0.6–0.7 mm anthers (early meiosis), when MEL1 and ZEP1 proteins are evidently expressed. Wes signals for both MEL1 and ZEP1 proteins coincide with the predicted molecular masses, demonstrating that the anti-MEL1 and anti-ZEP1 antibodies detect MEL1/ZEP1 proteins. Red arrows indicate the molecular mass positions of the MEL1 and ZEP1 proteins.

Supplementary Figure 2

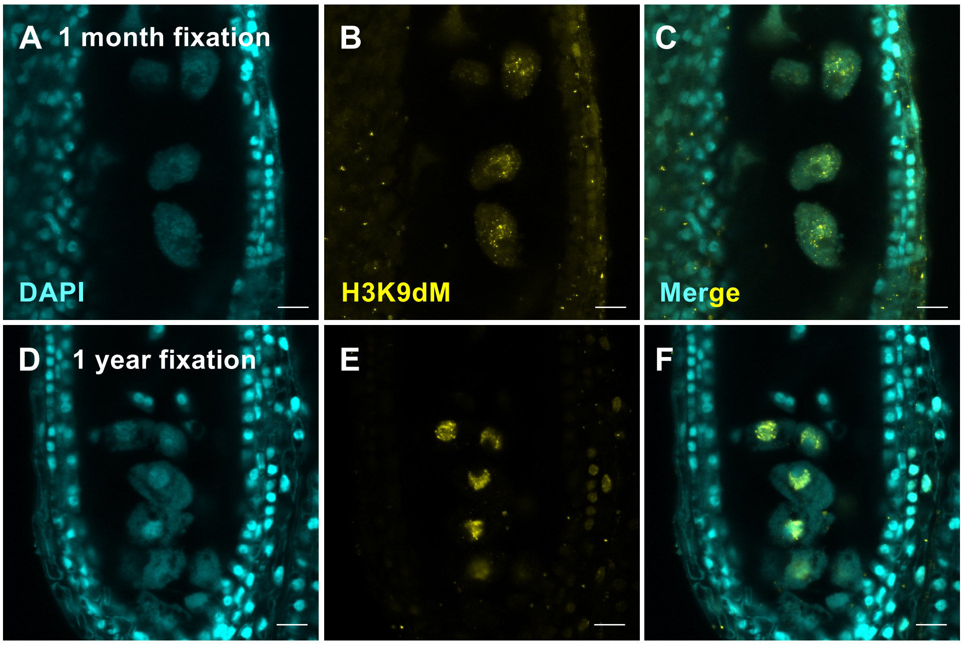

Figure S2. Comparison of H3K9 demethylation in anthers fixed one month and one year prior to 3D immunoimaging.
(**A and D**) DAPI signals (cyan) in 3D multiple immunoimaging using 0.5-mm-long whole anthers at pre- or early meiosis. (**B and E**) 3D multiple immunoimaging to detect histone 3 K9 dimethylation (H3K9dM; yellow). (**C and F**) Merged images of A and B, and D and E, respectively. H3K9dM is a general heterochromatin formation marker, and was observed in the nucleus of pollen mother cells of 0.5-mm anthers (**B and E**). This 3D immunostaining method also enables detection of the dot signals of H3K9dM in anthers fixed both one month previously (**A–C**) and one year previously (**D–F**). Scale bars, 10 μm.

Supplementary Figure 3

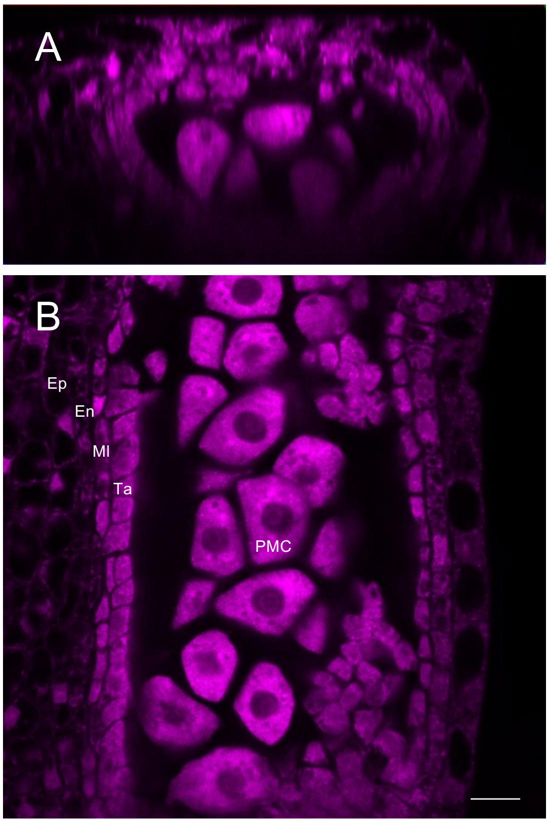

Figure S3. 3D histochemical imaging using whole anthers.

(**A**) Cross-section (X section) of a PI-stained a 0.5-mm-long anther. (**B**) Longitudinal section (Z section) of a PI-stained 0.5-mm-long anther. The four somatic layers, namely the epidermis (Ep), endothecium (En), middle layer (Ml), and tapetum (Ta), as well as pollen mother cells (PMC), were observed. Scale bar, 10 μm.

Supplementary Figure 4

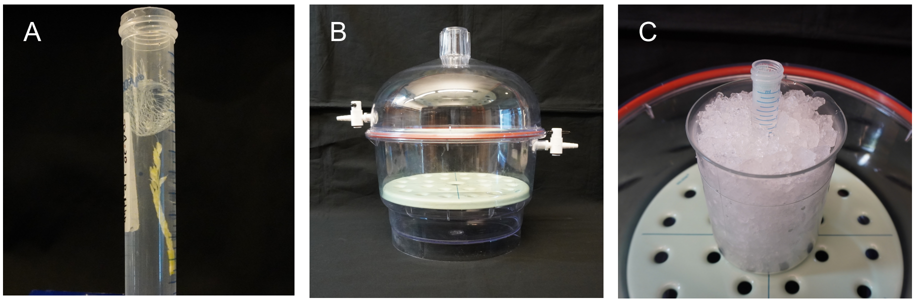

Figure S4. Equipment used for anther fixation.

(**A**) Inflorescences in 4% PFA fixative using a 15-mL plastic tube. (**B**) Vacuum desiccator for the fixation. (**C**) The 15-mL plastic tube with samples on ice during the fixation.
