# Supplementary material for: 3D multiple immunoimaging using whole male organs in rice
Source: Sci Rep. 2022 Sep 14;12:15426. doi: 10.1038/s41598-022-19373-4 (PMC9475021; doi:10.1038/s41598-022-19373-4)
Supplement: Supplementary file 5 — Supplementary Table S1. [file 41598_2022_19373_MOESM5_ESM.pdf]

Supplementary Table S1. Developmental stages of anthers

Stage	Anther (mm)	Germ cell development	Anther wall development	MEL1 localization	ZEP1 localization
stage 1	0.3–0.5	Premeiosis (primordial germ cell initiation)	Four layers (Ep, En, Ml, Ta)	Cytoplasm	-
stage 2	0.5–0.7	Early meiosis (Leptotene–Diplotene)	Four layers (Ep, En, Ml, Ta)	Cytoplasm	Filamentous signals on meiotic chromosomes
stage 3	0.6–0.8	Meiotic division	Ta differentiation	-	-
stage 4	0.8–0.9	Microspore	Ta programmed cell death Ml disappearance	N.D.	N.D.

Ep, epidermis; En, endothecium; Ml, middle layer; Ta, tapetum; -, no observation; N.D., no data
