# Supplementary material for: 3D multiple immunoimaging using whole male organs in rice
Source: Sci Rep. 2022 Sep 14;12:15426. doi: 10.1038/s41598-022-19373-4 (PMC9475021; doi:10.1038/s41598-022-19373-4)
Supplement: Supplementary file 6 — Supplementary Movie 1 legend. [file 41598_2022_19373_MOESM6_ESM.docx]

**Movie 1. 3D movie of MEL1/ZEP1 immunostaining using whole anthers.** Cyan signals indicate DAPI staining. Magenta signals indicate the indirect fluorescence of the MEL1 protein. Yellow signals indicate the indirect fluorescence of the ZEP1 protein. Sections of 0.65-mm-long anthers at early meiosis were used for 3D multiple immunoimaging. Scale bar, 10 μm.
